# Assessing early therapeutic drug monitoring of adalimumab as a predictor of treatment efficacy and immunogenicity in rheumatic diseases: “early therapeutic drug monitoring of adalimumab”

**DOI:** 10.1007/s10067-025-07307-0

**Published:** 2025-01-18

**Authors:** Patricia Ortiz-Fernández, Carles Iniesta-Navalón, Elena Urbieta-Sanz, Juan José Gascón-Cánovas

**Affiliations:** 1 Department of Hospital Pharmacy, Reina Sofia Hospital of Murcia, Murcia, Spain; 2 Clinical Pharmacokinetics and Applied Pharmacotherapy Group, Biomedical Research Institute of Murcia (IMIB-Pascual Parrilla), Murcia, Spain; 3 Department of Pharmacology School of Medicine, University of Murcia, Campus de Ciencias de la Salud, Murcia, 30120 Spain; 4 Department of Public Health, University of Murcia, Campus de Ciencias de la Salud, Murcia, 30120 Spain

**Keywords:** Adalimumab, Drug survival, Inflammatory rheumatic diseases, Therapeutic drug monitoring

## Abstract

**Introduction:**

Therapeutic drug monitoring (TDM) in inflammatory rheumatic diseases (RMDs) is gaining interest. However, there are unresolved questions about the best practices for implementing TDM effectively in clinical settings.

**Objective:**

The primary objective of this study was to evaluate whether early TDM of adalimumab predicts drug survival at 52 weeks in patients with RMDs. The secondary objective was to identify factors associated with pharmacokinetic failure and treatment discontinuation.

**Methods:**

A retrospective cohort study included patients aged ≥ 18 years with RMDs who initiated adalimumab therapy. Early TDM was performed within the first 26 weeks, and adalimumab trough levels (ATL) and anti-drug antibodies were measured. Drug survival was assessed at 52 weeks and defined as the time from adalimumab initiation to discontinuation for any reason. Multivariate analyses were conducted to identify factors influencing outcomes.

**Results:**

The study included 194 patients, of whom 56.7% exhibited ATL below the therapeutic range during the first 26 weeks. In the multivariate analysis, subtherapeutic concentrations were significantly associated with higher weight (OR = 1.02; *p* = 0.040) and ankylosing spondylitis diagnosis (OR = 3.68; *p* < 0.001). At 52 weeks, 43.8% of patients had discontinued adalimumab. Low ATL (< 1 µg/mL) was strongly associated with treatment discontinuation (OR = 7.31; *p* < 0.001), while concomitant methotrexate reduced this risk (OR = 0.46; *p* = 0.026).

**Conclusions:**

Early TDM of adalimumab predicts drug persistence and underscores its clinical relevance as a proactive tool to guide personalized treatment and reduce the risk of treatment failure. These findings highlight the importance of incorporating TDM into routine practice to optimize therapeutic outcomes.

**Table:** 

**Key Points** • *Early TDM of adalimumab in rheumatic diseases shows that low drug exposure predicts reduced drug survival at 52 weeks.* • *Approximately half of the patients exhibit low adalimumab exposure with the standard dose (40 mg every other week).* • *Body weight and methotrexate use significantly impact adalimumab levels.* • *Immunogenicity, found in 14.4% of patients with low ADL levels, underscores the need for early ADA detection to prevent non-response and discontinuation.*

## Introduction

Anti-tumor necrosis factor (anti-TNF) drugs, such as adalimumab (ADL), are a cornerstone in the treatment of inflammatory rheumatic musculoskeletal diseases (RMDs). ADL, a fully human monoclonal antibody that targets TNF, has demonstrated safety and efficacy across a range of IDRs including rheumatoid arthritis (RA), psoriatic arthritis (PsA), and ankylosing spondylitis (AS) [1–3].

Despite its benefits, many patients experience inadequate responses, either as primary failure (no initial response) or as secondary failure (loss of response over time), leading to drug discontinuation [2, 4]. The underlying mechanisms, of non-response may be pharmacodynamic, where the drug is present at optimal levels but fails to achieve the expected therapeutic effect because the primary inflammatory pathway is not TNFα-dependent; or pharmacokinetic, where drug levels are lower than desired due to immunological factors or other causes, such as increased drug clearance [5–7].

There is a known direct relationship between the plasma concentrations of anti-TNF drugs and their therapeutic efficacy, and the development of anti-drug antibodies can negatively impact this efficacy [8–12]. TDM of biological drugs offers the potential to personalize the therapeutic approach in patients with RMDs [6, 7, 11, 13–15]. TDM enables the measurement of drug concentration in plasma, as well as the detection of anti-drug antibodies, and it is a useful tool in various clinical situations, such as loss of response to treatment, dose adjustment, adherence to treatment, and interpretation of side effects, thereby facilitating therapeutic decision-making [13–15].

Recent evidence supports the utility of TDM in inflammatory bowel disease [16–19], showing benefits in both induction and maintenance phases and across proactive and reactive approaches. In rheumatology, there is growing interest in monitoring biological drugs to assess blood concentrations and detect anti-drug antibodies. However, questions remain about how to optimally apply TDM in clinical practice [20–22].

A recent study by Jyssum et al. [11] demonstrated high variability in serum adalimumab levels among patients on standard doses and early development of anti-drug antibodies (ADAbs), highlighting the potential role of TDM. Recently, the European Alliance of Associations for Rheumatology (EULAR) recommends considering early measurement of biopharmaceutical blood concentrations, within the first 3 months of treatment, to predict future efficacy [20]. Observational studies have investigated the predictive value of early biologic drug concentrations for subsequent clinical response [7, 11, 12, 23]. In patients treated with ADL, subtherapeutic blood concentrations at week 4 of treatment were associated with lack of clinical response at week 12 in spondyloarthritis [23], and subtherapeutic concentrations at week 12 were predictive of lack of response at week 52 [12]. Despite the findings, the implementation of TDM in daily clinical practice remains limited and lacks clear guidance on its application.

The primary objective of this study was to assess the potential of early TDM of ADL, measured within the first 26 weeks of treatment, as a predictor of drug survival over the first year in patients with RMDs. Secondary objectives included the identification of factors associated with pharmacokinetic failure and treatment discontinuation.

## Methods

### Study design and patient cohort

A retrospective observational cohort study was conducted at a regional reference hospital in Murcia, attending a population of 200,892 subjects. Patients were eligible for inclusion if they were ≥ 18 years; had a confirmed diagnosis of RA, PsA, or AS by a rheumatologist; and initiated treatment with ADL between October 2015 and January 2024. Additionally, patients required TDM data collected within the first 26 weeks of treatment. Exclusion criteria included insufficient clinical data (e.g., missing diagnostic, or treatment history information) or pharmacokinetic data (e.g., missing date of the last dose or recent missed doses within the last 2 months), loss to follow-up, and dosing regimens other than 40 mg of adalimumab administered subcutaneously every other week. The study received approval from the ethical research committee, CEIm Hospital Virgen de la Arrixaca (registration number 2024–13-HCUVA).

### Data collection

The study population was identified using electronic prescription records from the hospital’s outpatient Pharmacy Department. These records included detailed information on the type of biological therapy administered, treatment regimen, and demographic and clinical characteristics. Data collected encompassed sex, age, body weight, diagnosis, prior biological or disease-modifying antirheumatic drug (DMARD), specifics regarding biosimilar adalimumab or reference therapy, duration of treatment, and reason for treatment discontinuation.

### Sample extraction and processing

Serum samples were extracted at the hospital's pharmacy service for subcutaneously administered drugs. Adalimumab trough levels (ATLs) samples, taken immediately before the next scheduled dose, were collected once patients reached a steady-state condition, specifically from 12 weeks after the initiation of treatment up to 26 weeks. Prior to analysis, samples were centrifuged at 1500 g for 8 min, and two serum aliquots were prepared from each sample. These aliquots were stored frozen at − 20 °C and processed within a maximum of 30 days from the extraction date.

### Analytical determination

The analytical determinations were conducted at the Clinical Pharmacokinetics Unit of the hospital’s Pharmacy Service. The ELISA assay was performed using Promonitor® kits (Grifols®) following the manufacturer’s protocols. For the detection ATL, serum samples were diluted 1:400. With this dilution, the measurement range for ADL concentrations was between 0.52 and 24 µg/mL. For the detection of anti-adalimumab antibodies (ADAs), serum samples were used undiluted (1:1) and diluted 1:10. This method is a sensitive assay that cannot detect ADAs in the presence of ADL. For ELISA, the lowest quantification limits for ADAs were 3.7 AU/mL and results were considered positive when titers exceeded 10 AU/mL for ADAs. Antibodies were determined only when ADL trough levels were < 1 μg/mL.

### Endpoints

#### Pharmacokinetic outcomes

The primary endpoint of this study was to determine the percentage of patients achieving adequate ADL exposure during early TDM. This early monitoring was defined as trough-level sampling conducted between weeks 12 and 26 from the initiation of adalimumab treatment. Optimal ATL cutoff values were defined as ≥ 5 µg/mL for RA and PsA, and ≥ 8 µg/mL for AS during the maintenance phase. Additionally, the development of ADAs was monitored throughout the study period.

#### Drug survival outcomes

Drug survival was assessed at 52 weeks and was defined as the time interval from the initiation of ADL treatment to discontinuation for any reason. Reasons for discontinuation were classified as lack of response (including poor or partial response), immunogenicity, adverse events, surgery, and patient decision.

### Statistical analysis

Categorical data are presented as absolute numbers and percentages, while continuous variables are expressed as mean or median values with measures of variability, such as standard deviation (SD) or interquartile range (IQR). The Mann–Whitney *U* test was used to compare continuous variables, and Pearson’s chi-squared test was applied for categorical variables.

To analyze factors associated with adequate ADL exposure during early TDM, ADL treatment discontinuation, and immunogenicity, a univariate and multivariate logistic regression analysis was conducted using a stepwise forward selection method. Variables included in the multivariate model were selected based on their statistical significance in the univariate logistic regression analysis. Survival curves at 52 weeks, stratified by adequate ADL exposure, were generated using the Kaplan–Meier estimator, and the log-rank test was employed to compare ADL survival between groups.

Receiver operating characteristic (ROC) curve analysis was employed to determine the optimal cut-off for ATL associated with treatment persistence at week 52. The cut-off value was selected based on the maximum combined sensitivity and specificity. A *p*-value < 0.05 was considered statistically significant. Statistical analysis was performed using SPSS for Windows version 23.0 (IBM Corp., Armonk, NY, USA).

## Results

### Patient selection and baseline characteristics

A total of 205 patients initially met the inclusion criteria. However, 11 patients were excluded due to loss to follow-up (*n* = 4), administration of adalimumab at dosing regimens other than 40 mg every other week (*n* = 5), and insufficient pharmacokinetic data (*n* = 2). Therefore, 194 patients were included in the final analysis, of whom 114 were females (58.8%). The mean age at the initiation of ADL treatment was 51.2 years (SD, 11.7). Diagnoses included 74 patients with RA (38.1%), 57 with PsA (29.4%), and 63 with AS (32.5%). Among the RA patients, 40 (53.6%) tested positive for RF, and 45 (61.1%) were positive for anti-CCP antibodies. Statistically significant differences were observed in the proportion of females between the RA and AS groups, with a higher percentage of females in the RA group. Most patients, 189 out of 194 (97.4%), were receiving treatment with biosimilar adalimumab, with 110 patients (56.7%) on monotherapy. Overall, methotrexate was used in 31.4% of the study population. Its use was significantly higher among RA patients (44.6%) and lower in AS patients (12.7%) (*p* < 0.001). A total of 29 patients (14.9%) had prior exposure to biological treatments, primarily etanercept (11.9%) and infliximab (3.1%). The baseline characteristics of the population of the study are shown in Table 1.

**Table 1:** Baseline characteristics and group differences

	Total		Rheumatoid arthritis		Psoriatic arthritis		Ankylosing spondylitis		*p*
	*N*	*(%)*	*N*	*(%)*	*N*	*(%)*	*N*	*(%)*	
Patients	194	100	74	38.1	57	29.4	63	32.5	
Age (mean ± SD) years	51.2 ± 11.7		50.8 ± 13.3		51.8 ± 9.3		51.7 ± 11.6		0.832
Weight (mean ± SD)	77.2 ± 16.9		76.5 ± 15.9		76.0 ± 18.9		79.6 ± 16.0		0.495
> 65 years	25	12.9	11	14.9	5	8.8	9	14.3	0.541
Female	114	58.8	51	68.9	35	61.4	28	44.4	0.013
Rheumatoride factor positive, *n* = 142	38	19.6	37	53.6	0	0	1	2.7	< 0.001
Anti-CCP antibody positive, *n* = 81	34	17.5	33	61.1	0	0	1	6.3	< 0.001
HLA-B27 positive, *n* = 98	17	8.8	2	10	1	3.8	14	26.9	0.025
Adalimumab biosimilar	189	97.4	73	98.6	57	100	59	93.7	0.063
Previous TNF-inhibitors	29	14.9	15	20.3	4	7	10	15.9	0.105
One previous biological drug	22	11.3	13	17.6	1	1.75	8	12.7	0.026
≥ 2 previous biological drugs	6	3.1	1	1.4	3	5.26	2	3.17	
Etanercept	23	11.9	11	14.9	4	7	8	12.7	0.375
Infliximab	6	3.1	1	1.4	3	5.3	2	3.2	0.439
Certolizumab	2	1	1	1.4	1	1.8	0	0	0.600
Adalimumab	1	0.5	0	0	0	0	1	1.6	0.352
Golimumab	1	0.5	1	1.4	0	0	0	0	0.443
DMARD concomitant	84	43.3	43	58.1	24	42.1	17	27	< 0.001
Methotrexate	61	31.4	33	44.6	20	35.1	8	12.7	< 0.001

*CCP* cyclic citrullinated peptide, *DMARD* disease-modifying antirheumatic drug, *HLA* human leukocyte antigen, *TNF* tumor necrosis factor

### Adalimumab trough levels TDM

**Fig. 1:**
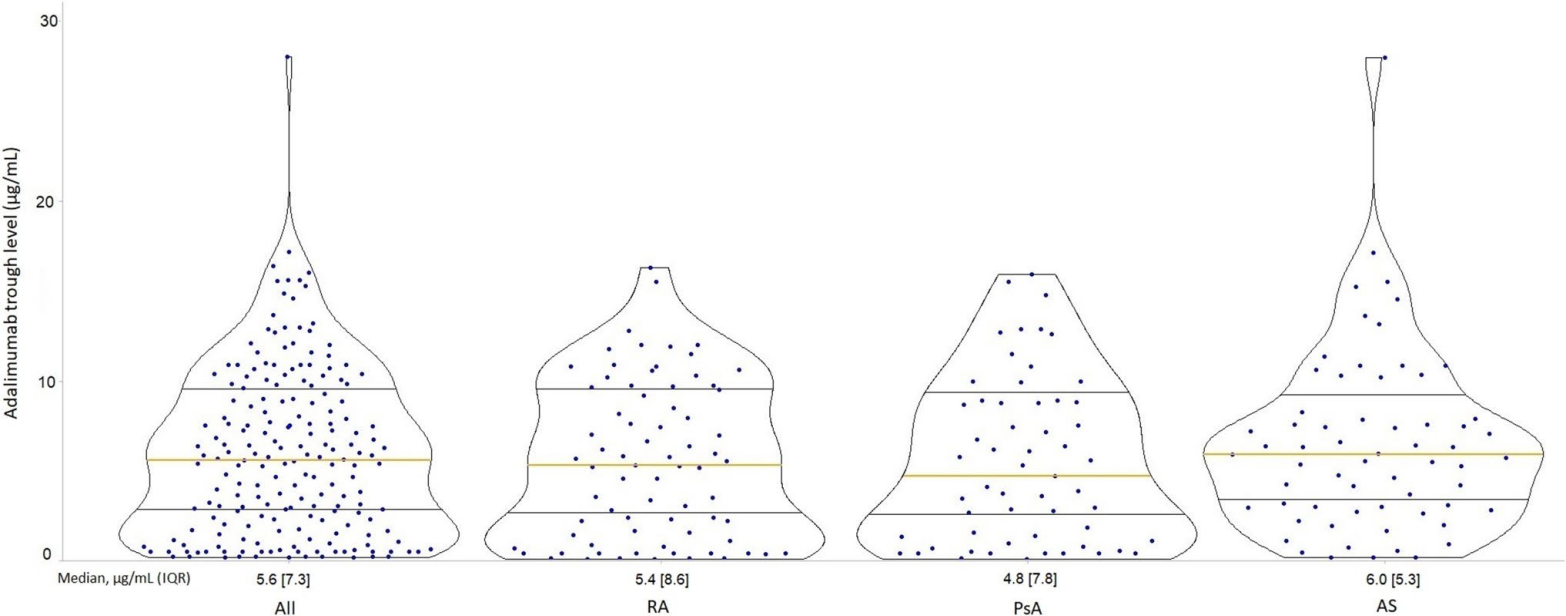
Adalimumab trough levels at early TDM. Violin plot displaying the probability density of trough levels across participants, smoothed by a kernel density estimator. Each data point represents an individual participant; the solid yellow line indicates the group median, and the black lines represent percentiles

Adalimumab trough levels were evaluated in the cohort of 194 patients during the initial 26 weeks of treatment. Of these, 99 patients (51%) had ATL measurements performed within the first 12 weeks. The median serum ATL during the first 26 weeks of treatment was 5.6 µg/mL (IQR, 7.3), showing no significant differences across diagnostic groups (*p* = 0.456) (Fig. 1). A total of 110 patients (56.7%) exhibited ATL below the therapeutic range during the first 26 weeks of treatment, with 38 of these patients (34.5%) presenting serum levels below 1 µg/mL. Subtherapeutic ATL was most frequently observed in patients with AS (74.6%), followed by those with PsA (50.9%) and RA (45.9%) (*p* < 0.001).

The results of the univariate analysis showed significant associations between subtherapeutic ATL and the following variables: AS (OR = 3.17 [95% CI, 1.63–6.15]; *p* < 0.001), RA (OR = 0.49 [95% CI, 0.27–0.88]; *p* = 0.018), weight (OR = 1.02 [95% CI, 1.00–1.04]; *p* = 0.021), and concomitant methotrexate (MTX) use (OR = 0.48 [95% CI, 0.26–0.88]; *p* = 0.018). In the multivariate analysis, subtherapeutic concentrations remained significantly associated with weight (OR = 1.02 [95% CI, 1.00–1.04]; *p* = 0.040) and AS (OR = 3.68 [95% CI, 1.80–7.53]; *p* < 0.001). Table 2 shows the factors associated with ATL below the therapeutic range during the first 26 weeks.

**Table 2:** Factors associated with concentrations below therapeutic range in the first 26 weeks (*n* = 110)

		Univariant		Multivariant	
	*N* (%)	OR [CI 95%]	*p*	OR [CI 95%]	*p*
Female	64/114 (56.1)	0.94 [0.53–1.68]	0.851		
≥ 65 years	15/25 (60.0)	1.17 [0.49–2.75]	0.721		
Weigth	-	1.02 [1.00–1.04]	0.021	1.02 [1.00–1.04]	0.040
Ankylosing spondylitis	47/63 (74.6)	3.17 [1.63–6.15]	< 0.001	3.68 [1.80–7.53]	< 0.001
AS HLA-B27 positive	9/14 (64.3)	0.48 [0.12–1.84]	0.279		
Rheumatoid arthritis	34/74 (45.9)	0.49 [0.27–0.88]	0.018	0.77 [0.37–1.59]	0.489
RA seropositive	14/37 (37.8)	0.57 [0.22–1.48]	0.250		
Psoriatic arthritis	29/57 (50.9)	0.71 [0.38–1.33]	0.291		
Adalimumab biosimilar	107/189 (56.6)	0.87 [0.14–5.32]	0.880		
Previous TNF-inhibitors	20/29 (68.9)	0.54 [0.23–1.25]	0.148		
Methotrexate	27/61 (44.3)	0.48 [0.26–0.88]	0.018	0.59 [0.30–1.17]	0.131

*RA* rheumatoid arthritis, *AS* ankylosing spondylitis, *HLA* human leukocyte antigen

### Drug survival at 52 weeks and causes of discontinuation

Within the first year of treatment, 85 of 194 patients (43.8%) discontinued adalimumab. The primary reasons for discontinuation included lack of response in 40 patients (47.1%), immunogenicity in 28 patients (32.9%), adverse events in 8 patients (9.4%), a combination of lack of response and adverse events in 7 patients (8.2%), surgery in 1 patient (1.2%), and patient decision in 1 patient (1.2%).

**Fig. 2:**
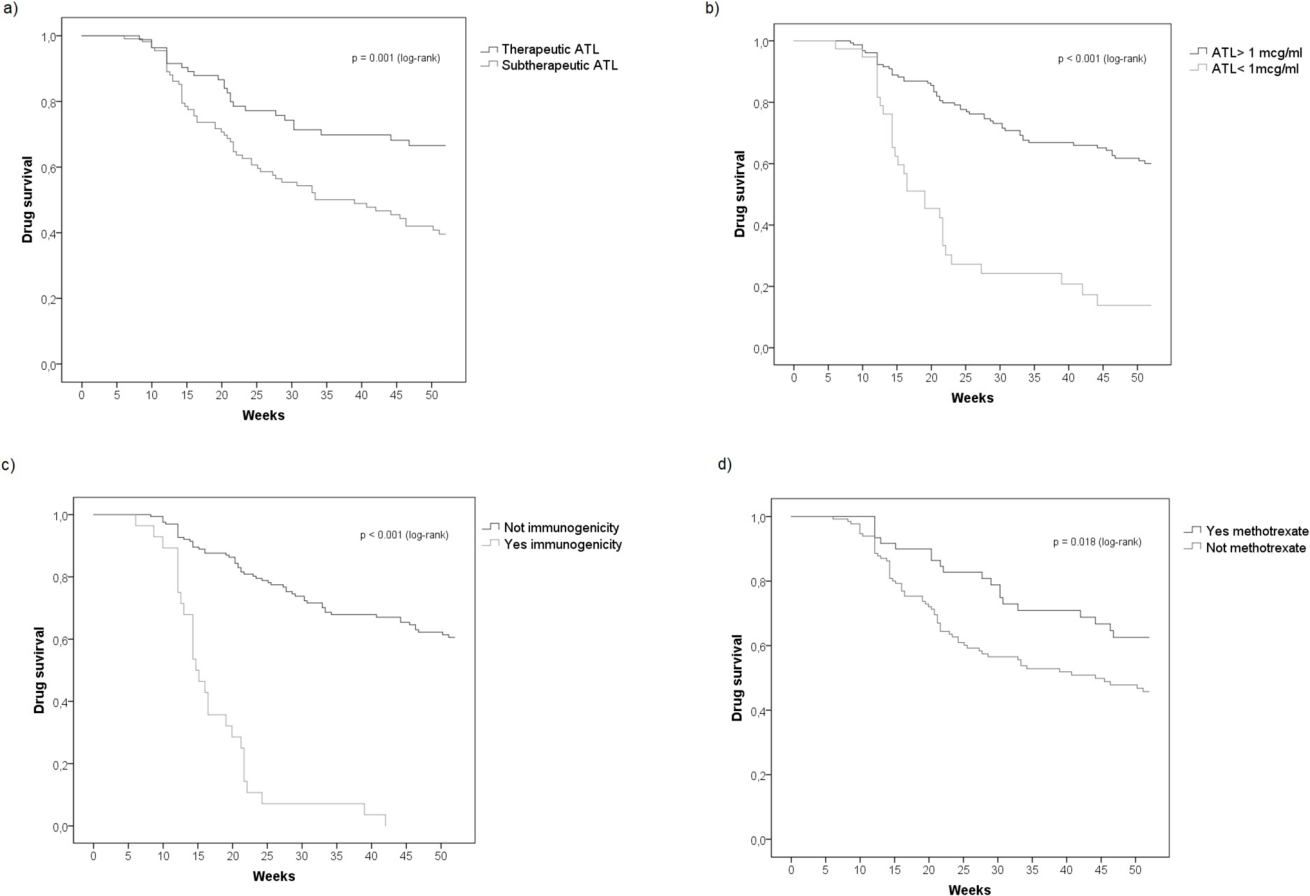
Drug survival curves stratified by the following: therapeutic versus subtherapeutic ATL levels (**a**); ATL < 1 µg/mL compared to levels ≥ 1 µg/mL (**b**); presence or absence of anti-adalimumab antibodies (immunogenicity) (**c**); and concomitant methotrexate (d)

The median follow-up period was 32.9 weeks (IQR, 35.0). The information on drug persistence curves for ADL over the first 52 weeks, stratified by immunogenicity, concomitant MTX use, and ATL, is presented in Fig. 2. Univariate analysis showed that immunogenicity (OR = 7.26 [95% CI, 2.62–20.14]; *p* < 0.001), subtherapeutic ATL (OR = 2.83 [95% CI, 1.55–5.16]), and ATL below 1 µg/mL (OR = 6.88 [95% CI, 2.95–16.05]) were significantly associated with higher rates of ADL discontinuation. Conversely, concomitant MTX use (OR = 0.51 [95% CI, 0.24–0.96]) was associated with lower discontinuation rates. Multivariate analysis identified ATL below 1 µg/mL as the only factor significantly associated with discontinuation within the first year (OR = 7.31 [95% CI, 3.08–17.37], *p* < 0.001). Conversely, concomitant MTX use was associated with a significantly lower risk of treatment discontinuation (OR = 0.46 [95% CI, 0.23–0.91], *p* = 0.026) (Table 3).

**Table 3:** Discontinuation adalimumab at 52 weeks (*n* = 85)

	Univariant			Multivariant	
	*N* (%)	OR [CI 95%]	*p*	OR [CI 95%]	*p*
Female	54/114 (47.4)	1.42 [0.79–2.54]	0.234	-	-
≥ 65 years	14/25 (56)	1.75 [0.75–4.09]	0.188	-	-
Ankylosing spondylitis	26/63 (41.3)	0.85 [0.46–1.57]	0.620	-	-
AS HLA-B27 positive	3/14 (21.4)	0.27 [0.06–1.13]	0.064	-	-
Rheumatoid arthritis	32/74 (43.2)	0.96 [0.53–1.73]	0.900	-	-
RA seropositive	18/37 (48.6)	1.66 [0.63–4.32]	0.300	-	-
Psoriatic arthritis	27/57 (47.4)	1.22 [0.66–2.28]	0.520	-	-
Weigth	-	1.01 [0.99–1.03]	0.258	-	-
Adalimumab biosimilar	84/189 (44.4)	3.2 [0.35–29.17]	0.277	-	-
Previous anti-TNF drugs	17/29 (58.6)	0.49 [0.22–1.10]	0.081	-	-
Immunogenicity	22/27 (81.5)	7.26 [2.62–20.14]	< 0.001	1.79 [0.44–7.32]	0.416
ATL < 5/8 µg/mL	60/110 (54.5)	2.83 [1.55–5.16]	0.001	1.49 [0.75–2.93]	0.249
ATL < 1 µg/mL	30/38 (78.9)	6.88 [2.95–16.05]	< 0.001	7.31 [3.08–17.37]	< 0.001
Methotrexate	20/61 (32.8)	0.51 [0.27–0.96]	0.036	0.46 [0.23–0.91]	0.026

*ATL* adalimumab trough level, *AS* ankylosing spondylitis, *RA* rheumatoid arthritis

### Optimal adalimumab trough level cutoffs for drug survival at week 52

At week 52, the treatment discontinuation rate was observed at 43.2% (32/74) in RA patients. In this group, early TDM median ATL was higher among patients who maintained treatment (5.84 µg/mL [IQR, 7.18]) than those who discontinued (3.30 µg/mL [IQR, 9.31]); however, this difference was not statistically significant (*p* = 0.162). An ATL threshold of 4.68 μg/mL demonstrated limited predictive accuracy for treatment continuation in RA, with an AUC of 0.595 (95% CI, 0.45–0.74; *p* = 0.163), sensitivity of 64.3%, and specificity of 56.2%. In PsA patients, the discontinuation rate at week 52 was 47.4% (27/57). Early TDM median ATLs were significantly higher in patients who continued treatment (6.68 µg/mL [IQR, 5.74]) compared to those who discontinued (1.44 µg/mL [IQR, 7.00]; *p* = 0.006). For PsA, an ATL threshold of 4.11 μg/mL showed moderate predictive capability for ongoing treatment, yielding an AUC of 0.712 (95% CI, 0.57–0.85; *p* = 0.006), with sensitivity and specificity of 70.0% and 66.7%, respectively. In AS patients, treatment discontinuation at week 52 was reported in 41.3% (26/63). The median ATL in early TDM was 6.48 µg/mL (IQR, 5.91) in patients who maintained treatment versus 3.51 µg/mL (IQR, 5.40) in those who discontinued (*p* = 0.007). An ATL cutoff of 5.59 μg/mL produced an AUC of 0.700 (95% CI, 0.57–0.83; *p* = 0.007), suggesting moderate predictive value for treatment continuation with a sensitivity of 70.3% and specificity of 69.2%.

### Immunogenicity

Immunogenicity was detected in 28 patients (14.4%), all of whom had ATL below 1 µg/mL. The median serum level of ADAs was 101.0 UA/mL (IQR, 166), with a median time to appearance of 14.1 weeks (IQR, 7.5). Analyses of factors associated with immunogenicity within the first 52 weeks identified a significant association only with AS (OR = 0.30 (95% CI, 0.10–0.91); *p* = 0.026), indicating a lower likelihood of antibody formation in AS compared to other conditions. No significant associations were found for other key variables, including PsA, RA, concomitant methotrexate use, biosimilar adalimumab use, or treatment-naïve status (Table 4).

**Table 4:** Factors associated with immunogenicity (*n* = 28)

		Univariant	
	*N* (%)	OR [CI 95%]	*p*
Female	19/114 (16.7)	1.57 [0.67–3.69]	0.291
≥ 65 years	3/25 (12.0)	0.78 [0.22–2.82]	0.711
Ankylosing spondylitis	4/63 (6.3)	0.30 [0.10–0.91]	0.026
Rheumatoid arthritis	13/74 (17.6)	1.49 [0.66–3.34]	0.329
RA seropositive	5/37 (13.5)	0.70 [0.19–2.56]	0.592
Psoriatic arthritis	11/57 (19.3)	1.68 [0.73–3.87]	0.214
Weight	-	0.99 [0.96–1.01]	0.427
Adalimumab biosimilar	28/189 (14.8)	1.17 [1.10–1.25]	0.352
Previous TNF-inhibitors	7/29 (24.1)	0.46 [0.17–1.20]	0.107
Methotrexate	7/61 (11.5)	0.69 [0.28–1.73]	0.427

*RA* rheumatoid arthritis

## Discussion

In this retrospective study, we evaluated the clinical relevance of early TDM of ADL in patients with RMDs, specifically examining the variability in ADL exposure and its association with treatment persistence over the first year. These conditions share common challenges, such as pharmacokinetic variability, immunogenicity, and treatment response, highlighting the relevance of TDM to optimize adalimumab therapy across inflammatory diseases. Our findings indicate that only half of the patients achieved therapeutic ADL concentrations within the first 26 weeks, and this reduced exposure was associated with lower treatment persistence at 52 weeks. These results underscore the importance of early TDM to optimize treatment outcomes in clinical practice.

Our study found that more than half of the patients exhibited ATL below the therapeutic range within the first 26 weeks, with low exposure most pronounced in patients with AS, followed by those with PsA and RA. Notably, approximately one-fifth of the total patients (38 out of 194) had concentrations below 1 µg/mL. The observed differences in exposure between AS, PsA, and RA can be largely attributed to the therapeutic ranges established for each condition. For AS, an effective ATL is generally defined as 8–12 µg/mL [23], which is higher than the 5–8 µg/mL [8, 12] range recommended for RA and PsA. This distinction emphasizes the importance of diagnosis-specific TDM thresholds in achieving optimal therapeutic outcomes across different RMDs populations. Our findings align with prior research identifying patient-specific factors that impact ADL exposure.

Consistent with existing studies [8, 11, 12, 24], we observed that body weight was associated with subtherapeutic ADL levels, suggesting that individualized dosing strategies may be warranted to enhance therapeutic drug exposure and clinical response. Jani et al. [12] reported that a BMI cutoff of ≥ 30 kg/m^2^ was significantly associated with lower drug levels in adalimumab-treated patients. High BMI may contribute to reduced therapeutic response, not only due to bioavailability and distribution volume issues that lower drug levels, but also through the pro-inflammatory effects of adipose tissue, which may increase the target load [25]. The study by Ternant et al. [24] aimed to evaluate the pharmacokinetics of adalimumab in patients with RA, with a specific focus on identifying factors that influence drug clearance. Among their findings, they observed that higher body weight was associated with increased clearance of adalimumab, indicating that heavier RA patients may require adjusted dosing to achieve optimal therapeutic levels.

Previous studies have shown that methotrexate positively influences the pharmacokinetics of ADL [5, 11, 12, 26]. Our findings align with those of Jyssum et al. [11], who reported higher ADL levels in RA and PsA patients receiving concomitant MTX. In our study, no significant association was observed in AS, likely due to the low number of AS patients on MTX, limiting the assessment of its impact on ADL exposure in this group. In line with these findings, Martinez’s [26] study demonstrated a dose-dependent effect of MTX, with patients receiving doses ≥ 15 mg/weekly were five times more likely to have circulating TNFi in serum compared with patients receiving monotherapy.

In our study, immunogenicity was observed in 14.4% of patients. The variability in immunogenicity rates reported across studies (10–60%) is primarily due to differences in assay methodologies. Drug-tolerant assays typically report higher ADAs prevalence by detecting antibodies despite circulating drug, whereas drug-sensitive assays, such as the one used in our study, detect ADAs only when drug levels are low or absent, potentially resulting in lower immunogenicity rates [27].

Our findings are consistent with those of Jyssum et al. [11], who reported that 10% of patients developed ADAs within the first 3 months of adalimumab treatment using a drug-sensitive assay. In that cohort, the majority of ADAs-positive patients exhibited levels above 50 mg/L, a threshold associated with clinically significant, neutralizing, and persistent antibodies. Bartelds et al. [9] demonstrated that two-thirds of ADAs-positive RA patients developed antibodies within the first 28 weeks, which correlated with lower serum adalimumab levels and an increased rate of treatment discontinuation. These findings underscore the importance of proactive TDM early in treatment, when ADAs are most likely to emerge. Early detection of ADAs could be crucial for identifying causes of non-response, allowing timely pharmacotherapeutic adjustments and potentially preventing treatment discontinuation due to immunogenicity.

The prevalence of immunogenicity in our study was lower in patients with AS than in those with RA and PsA, aligning with findings from the NOR-DRUM trial [28], which reported similar results for infliximab. One factor contributing to the higher ADAb rates observed in RA may be elevated levels of B-cell activating factor (BAFF), a cytokine involved in RA pathogenesis and associated with increased ADAb formation against TNF inhibitors. This suggests that BAFF may play a role in promoting immunogenicity in RA [5, 29, 30]. Although prior studies suggest that methotrexate use reduces ADAs formation [5], our results did not show a statistically significant association between methotrexate and ADAs presence. However, methotrexate was associated with higher rates of treatment continuation and prevention of low serum drug levels, reinforcing its role in sustaining therapeutic efficacy.

Our study’s results regarding treatment persistence align with previous findings [11, 31], which report that higher ADL levels correlate with improved long-term treatment continuation in RMDs. Notably, the ADL thresholds identified in our cohort—4.11 µg/mL for PsA and 5.59 µg/mL for AS—are lower than those reported in previous studies [8, 11, 12, 23, 32]. This difference may be attributed to our use of persistence as an outcome, which is a less stringent measure compared to clinical remission. In RA, we were unable to establish a reliable threshold due to the high variability in ADL levels among patients.

This elevated variability in ATL underscores the clinical value of early TDM of ADL as a proactive measure to personalize the management of RMDs. Our findings not only highlight the variability in adalimumab exposure among patients with RMDs but also reinforce the importance of considering that fixed subcutaneous dosing can be a limiting factor, particularly in populations with diverse pharmacokinetic profiles (e.g., body weight and elevated inflammatory burden). Identifying subtherapeutic ADL levels during the initial phase of treatment allows for targeted interventions, such as dose adjustments or the introduction of MTX, which can help mitigate the risk of immunogenicity and improve drug retention. Furthermore, early detection of low ADL levels enables timely modifications to therapy, potentially enhancing patient adherence and optimizing long-term treatment outcomes.

The limitations of this study include its retrospective nature; however, TDM data were collected prospectively, which minimized the amount of missing data. Additionally, the sample size was insufficient to provide adequate statistical power for subgroup analyses, limiting the ability to detect specific effects within subpopulations. Furthermore, we did not have access to disease activity indices, which would have enabled the determination of ADL thresholds based on clinical activity levels, potentially refining therapeutic targets for better precision in treatment. Nonetheless, we consider drug survival to be a suitable indicator of drug response.

Despite these limitations, our findings are significant as they provide evidence supporting the utility of early TDM. Our results emphasize that more than half of the patients received ineffective therapy during the initial treatment period. This, along with the early onset of immunogenicity, underscores the need for improved strategies to optimize drug dosing and patient follow-up, particularly within the framework of personalized medicine. Early detection of subtherapeutic ADL levels can serve as a predictor of therapeutic failure, highlighting the importance of proactive TDM in clinical practice to enhance patient outcomes.

Further prospective research is warranted to evaluate the impact of TDM-guided interventions on long-term treatment efficacy and immunogenicity in RMD patients. Additionally, examining the effects of adjusted dosing regimens based on ADL levels may help establish more precise guidelines for TDM, enabling personalized treatment approaches that consider individual pharmacokinetics and disease characteristics. The NOR-DRUM studies [33, 34] assessed the utility of proactive TDM in infliximab treatment for immune-mediated inflammatory diseases, examining both induction [33] and maintenance [34] phases. While no significant remission differences were found at 30 weeks during induction, proactive TDM may benefit patients at high risk for anti-drug antibodies. In the maintenance phase, proactive TDM reduced the likelihood of disease worsening at 52 weeks compared to standard therapy, suggesting potential benefits despite sample size limitations that restrict subgroup analyses.

In conclusion, our study underscores the clinical relevance of early TDM of ADL in patients with inflammatory rheumatic diseases, demonstrating that low ADL levels during the initial treatment period are associated with reduced treatment persistence. These findings emphasize the need for further prospective studies and clinical trials to evaluate TDM's impact on long-term efficacy and immunogenicity in this patient population.

## Data Availability

The datasets presented in this study are available from the corresponding authors with reasonable requests.
